# Recent advances in managing Peyronie’s disease

**DOI:** 10.12688/f1000research.20557.1

**Published:** 2020-05-20

**Authors:** Asrit Babu, Oliver Kayes

**Affiliations:** 1 Department of Urology, Leeds Teaching Hospitals, Leeds, UK

**Keywords:** Peyronie, collagenase, traction therapy, fibrosis, treatment, Peyronie’s disease, urology, erectile dysfunction, non-surgical treatment

## Abstract

Treating men with Peyronie’s disease remains a challenging problem facing clinicians working across urology and sexual medicine fields. Patients can often be left disappointed by current treatment paradigms, and an overall lack of suitable molecular targets has limited the options for novel, effective medical therapy. Managing men with Peyronie’s disease often involves careful counselling alongside multifaceted and possible combination treatments to help improve symptoms whilst ameliorating potential side effects of therapy. We review the latest medical literature and evidence in the contemporary management of Peyronie’s disease.

## Background

Peyronie’s disease (PD) is a relatively common progressive fibrotic disorder that affects the tunica albuginea. Traditionally, men will present with a new penile curvature, which may be associated with pain in the acute phase. Prevalence is reported to be in the region of 0.4–20%, with manifestation typically in the fifth decade of life ^1– 3^. PD may also be frequently associated with erectile dysfunction, penile shortening, psychological distress, and a palpable plaque, which is typically found on the dorsal aspect of the penile shaft. In some men, the penile curvature is severe enough to impair penetrative sexual intercourse.

The natural history of PD is divided into the active and quiescent (chronic or stable) phases. During the active phase, patients may report penile pain with a changing deformity (1–6 months). In contrast, the stable phase is generally associated with a painless and stable penile deformity (6–18 months).

Observational studies have shown that the majority of patients with PD will eventually have a stable curvature (40–47%), with a similar proportion showing progression (40–48%) and a much smaller group spontaneously improving (12–13%) ^4, 5^. PD affects men principally between the ages of 40 and 60 and is associated with diabetes mellitus, Dupuytren’s contracture, and plantar fascial contracture (Ledderhose disease). There may be a preceding history of trauma.

The most common hypothesis regarding aetiology of the disease involves repeated minor microvascular trauma during intercourse resulting in intratunical bleeding and subsequent inflammation and fibrosis. Interestingly, we have seen PD in sexually naïve men who have never had penetrative intercourse. This may not refute the hypothesis but points further to a multifactorial process. Transforming growth factor beta (TGF-β) is thought to exacerbate the erratic healing response. Histologically, there is excessive connective tissue, increased cellularity, and random orientation of collagen fibres within the Peyronie’s plaque. Subsequently, the dysfunctional tunical tissue restricts normal expansion of the underlying corpus cavernosum, creating the observed curvature. There may be flaccidity distal to the lesion with or without a waisting (hourglass) deformity or rotation observed in more severe cases.

Surgical correction has historically been the mainstay of treatment but can harbour significant morbidity and in some series is related to poorer patient-reported outcomes. Patient morbidity from surgery includes haematoma and infection in the early post-operative period, with penile shortening, recurrent/residual curvature, glans hypoesthesia, and delayed erectile dysfunction manifesting later. In the majority of published case series, the main reasons for poor patient-reported outcomes tend to relate to the presence of residual curvature, overt penile shortening, and erectile dysfunction, with dissatisfaction rates ranging from 27–45% in those undergoing plication or plaque excision and grafting ^6, 7^. As a result of this, there has been extensive research into non-surgical interventions that can potentially stabilise or improve penile curvature without the morbidity associated with surgery.

The aim of this review is to summarise the most recent advances in PD treatments, including surgical techniques, non-surgical interventions, and basic science updates. A review of the literature from 2017 onwards (24 months) was conducted by performing a MEDLINE® search of publications using the keyword “Peyronie”. In the absence of any new data for specific treatments or to consolidate the evidence around newer therapies, we have included older key papers for the readership’s general information.

## Basic science

The most accepted pathophysiological aetiology of PD is that of microtrauma. This results from repeated injury resulting in an inflammatory response that promotes the formation of fibrous plaques through mediators such as TGF-β. There are very few studies that are able to show a clear correlation between the histological findings of trauma and Peyronie’s plaques in human histological specimens ^8, 9^. El-Sakka *et al.* verified that TGF-β is strongly expressed in PD histology in a rat model, leading to an inflammatory process and fibrosis ^10^. De Rose *et al.* conducted a prospective observational study comparing the histological and ultrastructural changes seen in patients undergoing plaque excision for PD (in the absence of trauma) and those undergoing plaque excision for a history of penile fracture. The results showed the two groups had very similar collagen deposition, cellular composition, and extracellular matrix, in keeping with the proposed aetiology of microtrauma being the underlying cause of PD ^11^.

## Stem cell therapy

Stem cells are undifferentiated cells that are capable of self-renewal and differentiation, promoting the repair of tissues via their immunomodulatory and anti-inflammatory action. Adipose-derived stem cells (ADSCs) are used most widely owing to their abundant tissue source and ease of isolation. There is currently limited evidence for the clinical use of stem cells in PD, with all studies restricted to rat models. Milenkovic, Albersen, and Castiglione reviewed the current evidence, which was limited to only four pre-clinical studies using ADSCs ^12^. Overall, these data support positive effects through differing proposed mechanisms of action, including reducing collagen and elastin deposition, reducing fibrosis, and increasing myofibroblast apoptosis ^13– 16^. Further clinical studies are needed to confirm the efficacy of stem cell therapy for PD in humans.

## Pharmacotherapy

No oral pharmacotherapy is recommended by the American Urological Association (AUA) or the International Consortium of Sexual Medicine (ICSM) because of the lack of robust evidence. In contrast, the European Association of Urology (EAU) suggest that potassium para-aminobenzoate (Potaba) may result in a significant reduction in curvature, plaque size, and pain ^17– 19^.

Generally, the EAU, AUA, and ICSM guidelines have similar recommendations but differ on a few key points. These comparative points are highlighted in Table 1. Unfortunately, it is beyond the scope of this review to further delineate any methodological or reporting differences used between these different guidelines panels that might generate further discussion regarding monotherapy or combination treatments. Suffice to say, given the relative paucity of high-quality evidence in this area, it is reasonable to concur that current oral monotherapies are universally considered to be of limited clinical use as a medical tool to directly reverse or modify PD outcomes in patients, either for symptom relief or to modify or improve longer-term functional outcomes.

**Table 1.** Non-operative management of PD: summary of EAU, AUA, and ICSM guidelines ^17– 19^.

Therapy	EAU (2015)			AUA (2015)			ICSM (2016)		
	Advised	Not advised	LoE	Advised	Not advised	LoE	Advised	Not advised	LoE
**NSAID**			-	✓		-			-
**Potaba**	✓		C		×			×	B
**Vitamin E**		×	B		×	B		×	B
**Tamoxifen**		×	B		×	B		×	B
**Carnitine**		×	C		×	B		×	B
**Pentoxifylline**		×	C		×	B		×	B
**Colchicine**		×	C		×	B			-
**Collagenase (I/L)**	✓		B	✓		B	✓		B
**Verapamil (I/L)**	✓		C	✓		C	✓		C
**Interferon (I/L)**	✓		C	✓		C	✓		B
**Steroids (I/L)**		×	B		×	-		×	
**Verapamil gel**	✓		C		×			×	B
**Verapamil and steroids (I/O)**	✓		C		×	-		×	B
**ESWT**	✓	×	C		×	B		×	B
**Mechanical**	✓		C		×	-	✓		C

AUA, American Urological Association; EAU, European Association of Urology; ESWT, extracorporeal shockwave therapy; ICSM, International Consortium of Sexual Medicine; I/L, intralesional; I/O, iontophoresis; LoE, level of evidence; NSAID, non-steroidal anti-inflammatory drug; PD, Peyronie’s disease; Potaba, potassium para-aminobenzoate.

### Potaba

Two placebo-controlled randomised controlled studies (RCTs) have shown evidence that Potaba may reduce progression and pain ^20, 21^. Weidner *et al.* demonstrated that penile plaque size was stabilised in the Potaba group compared to placebo. This subsequently reduced the progression of curvature in the treatment group when compared to placebo; however, it did not reduce any established curvature. This group did not demonstrate any evidence of improvement in pain relief. This is in contrast to the earlier RCT by Shah *et al.*, which demonstrated no evidence of improvement in curvature/plaque; however, it did show there was some improvement with regard to pain. Based on these two RCTs, Potaba currently is the only oral medication that has any recommendation for use within the EAU guidelines.

More recently, Potaba monotherapy was compared to combination therapy (tamoxifen, L-carnitine, and tadalafil) by Park *et al.* ^22^. Perhaps the most striking result was that two-thirds of the patients in the Potaba arm withdrew for various reasons, although treatment side effects were cited as the largest single factor. The study failed to show any statistically significant difference between the two treatment arms owing to the high dropout rate. Overall, the most clinically relevant conclusions from this study showed that the side effect profile of Potaba may lead to very poor compliance and a high discontinuation rate.

The potential adverse effects of treatment alongside the limited evidence of efficacy therefore limit the recommendation of Potaba use, which is why it is not recommended by the AUA or ICSM at the time of writing.

### Vitamin E and antioxidant therapy

Vitamin E is a fat-soluble, naturally occurring antioxidant that has previously been shown to improve pain, curvature, and erectile function scores (IIEF) and has been utilised in combination therapy with verapamil injections, non-steroidal anti-inflammatories, and herbal antioxidants, as reported in two recent randomised trials ^23, 24^.

The same authors have performed a further study in combination therapies using vitamin E. This case series was not randomised or controlled. A total of 141 patients were included in the study and assigned to one of five treatment groups that used varying amounts of antioxidant, analgesia, and injectable therapies including vitamin E, silymarin, Gingko biloba, topical diclofenac, and pentoxifylline injections. Throughout all groups, there was prevention in progression of disease. As the number of treatments increased, there was an improved outcome, particularly in IIEF score, reduced curvature, and improved quality of life/bother scores. The curvature improvement was minimal (4–6°) and likely clinically insignificant. In view of the study design not being randomised or controlled, the results must obviously be taken with caution ^25^.

### Phosphodiesterase-5 inhibitors and tamoxifen

A recent scientific study has investigated a new model to test potential medical therapies in PD. The team describe an *in vitro* model that mimics the cellular changes seen in PD. It is well understood that myofibroblasts play a large role in the remodelling of the extracellular matrix and the production of profibrotic mediators and inflammatory cytokines ^26, 27^. These cell lines have also been isolated in PD plaques ^28^. The authors were able to create a screening assay to assess the effectiveness of multiple treatments on the transformation of fibroblasts to myofibroblasts. Using this innovative model, they assessed the efficacy of 21 commonly studied oral therapies. The only compounds that proved to be effective on the fibroblast model included phosphodiesterase (PDE)-5 inhibitors and tamoxifen. These medications were then tested further in separate testing systems. The authors noted that each medication has the potential to prevent progression of disease in the active phase but is unlikely to reduce the plaque or curvature. They also identified that there was a synergistic effect with the two medications combined compared to either medication alone ^29^.

### Pentoxifylline

Pentoxifylline is a non-specific PDE inhibitor that also has some effect in reducing TGF-β tissue levels and therefore may have an antifibrotic role and thereby a therapeutic role in PD ^30^. The evidence of its efficacy is limited as a monotherapy; however, there is some evidence to suggest its benefit as a combination treatment.

Smith *et al.* investigated the effect of pentoxifylline monotherapy on plaque calcification and subjective improvement of the clinical condition. They studied 71 men in a cohort study, of whom 62 were treated with pentoxifylline and nine received no treatment. The study showed that 92% of patients treated with pentoxifylline compared to 44% with no treatment had stable or improved plaque calcification. There were no objective outcomes to suggest improvement in curvature; however, there was subjective patient-reported improvement, which makes it somewhat difficult to make any firm conclusions regarding its true clinical benefit ^31^.

A further cohort study conducted by Alizadeh *et al.* compared pentoxifylline with intralesional verapamil and a combination of both therapies. A total of 90 patients were enrolled into three treatment arms (n = 30) with no control group. They reported improvement in curvature, plaque size, pain, and erectile dysfunction in all groups. Outcome measures were not quantitative and therefore no conclusions about degree of curvature change can be made ^32^. Recently, an older double blind RCT conducted by Safarinejad in 2010 using pentoxifylline monotherapy has since been redacted because of statistical incongruities ^33^.

A study exploring pentoxifylline as part of a combination therapy has been recently completed by Ibrahim *et al.* ^34^; a total of 46 patients were included in this retrospective cohort study, which aimed to assess the effect of colchicine or pentoxifylline with penile traction therapy (PTT) (Andropenis® extender) on plaque size, degree of curvature, and penile Doppler parameters. Patients were assigned to oral pentoxifylline (n = 27) and colchicine (n = 18) with all advised to use PTT for 1 hour daily for a total of 6 months. The study reported an improvement in curvature of 14° (55.8° versus 41.4°) and also reported improvement in peak systolic velocity and reduction in plaque size. They found no statistically significant difference in the colchicine or pentoxifylline arm. The study has significant limitations in that there was no control group; therefore, it is possible that the improvement may have been purely spontaneous. It must also be noted that all patients received PTT, which potentially could explain why these parameters improved rather than as a result of any efficacy from the oral therapies. The efficacy of the Andropenis® extender is discussed further in this paper.

## Intralesional injection therapy

### Collagenase

Collagenase clostridium histolyticum (CCH) has been studied for use in PD in the experimental field since 1982 ^35^. Since 2013, CCH has been approved by the United States Food and Drug Administration as well as the European Medicines Agency. Treatment comes in the form of an injection of two collagenases, which act synergistically to cleave tropocollagen. Its use is recommended by the AUA for PD curvature between 30° and 90°, and the EAU guidelines currently advise a grade B recommendation; however, these guidelines precede the largest published RCT ^17^.

IMPRESS I and II were large RCTs comparing CCH and penile modelling against penile modelling and placebo with 417 and 415 patients included in the study, respectively. Exclusion criteria included any patients with an hourglass deformity, significant erectile dysfunction non-responsive to PDE-5 inhibitors, proximal plaques, and curvature outside 30–90°. The treatment protocol involved two injections of CCH (0.58 mg) 24 to 72 hours apart. This cycle regimen was then repeated up to four times, alongside penile modelling by the clinician at the time of injection as well as by the patient three times daily thereafter until review. Curvature improved by 17° and 9° (34% versus 18.2%) in the treatment and placebo arms, respectively. There was also similar improvement in IIEF scores (+1 and +0.4, respectively) as well as penile length (0.4 cm and 0.2 cm, respectively). PD questionnaire (PDQ) bother scores were also improved in the treatment group; however, one-third of the cohort did not complete the questionnaire at one or both measurement points, and the PDQ is still not validated psychometrically ^18, 36^.

Ralph *et al.* conducted a randomised study comparing CCH, modelling, and vacuum therapy to CCH and vacuum therapy alone. This small pilot study (n = 30) did not show any statistically significant difference between the two groups in terms of curvature improvement or patient-reported outcome measures. There was a clear improvement in curvature of 23.7° in the CCH, modelling, and vacuum therapy group compared to 23.3° in the group without penile modelling. The injection protocol was similar to that in the IMPRESS trial with the addition of vacuum therapy being initiated following the second injection twice daily throughout the remainder of the study. There was only clinician modelling performed, with no patient modelling performed in either arm of the study ^36, 37^.

A further study by this same group explored a modified injection protocol in a single-centre study of 53 patients alongside vacuum therapy during remodelling. The injection protocol consisted of three injections of 0.9 mg of CCH every 4 weeks. This was accompanied by manual modelling as well as vacuum therapy, and results showed equivalent outcomes to the IMPRESS trial with mean reduction in curvature of 17.3° (31.4%). This treatment regime reduced the need for multiple visits for injections and clinician modelling, which has the potential to lower costs and time for treatment ^38^. More recently, Capece and colleagues used the modified injection and remodelling protocol to perform a non-randomised, non-controlled multicentre study of 135 patients. They were able to reproduce the efficacy as described previously, with a mean reduction in curvature of 19° (42%). They did, however, note a slightly higher complication rate in the form of ecchymosis and haematoma when compared to the IMPRESS trial ^39^.

Ziegelmann *et al.* compared CCH and PTT versus CCH alone using a similar injection protocol to the IMPRESS trial with the addition of PTT for 3 hours per day. They found no significant difference in outcomes for CCH alone versus CCH and concomitant PTT. Overall curvature improvement was similar to that of the IMPRESS study, with a mean of 34% improvement. They reported poor adherence to the traction therapy with reducing compliance throughout the study, therefore likely limiting its effect. Ultimately, the authors felt that further RCT studies will be required to determine the role of traction therapy with CCH ^40^.

The latest published study in CCH treatment from an Italian group demonstrates a matched-pair comparison analysis of CCH in combination with sildenafil during the modelling phase. Using the same injection protocol as described in their previous study, they added sildenafil 25 mg BD 30–60 minutes prior to patient modelling. This was a small cohort study of 50 patients comparing the modified CCH protocol against CCH with the addition of sildenafil. They were able to show improved outcomes in curvature, IIEF scores, and PDQ scores, which were deemed to be statistically significant; however, the overall cohort was small. Curvature was improved by 25.6° in the CCH + sildenafil group compared to 17.4° in the group given CCH alone. This is clearly a promising outcome but needs further study in larger RCTs prior to any firm conclusions or recommendations being made ^41^.

CCH has clear robust RCT data showing definitive benefit in patients with PD and a penile curvature between 30° and 90°. There are numerous single-arm studies assessing variable treatment protocols that suggest that dosing adjustments can be made with associated accelerated treatment times and cost savings without compromising outcomes or safety parameters. It must also be noted that the IMPRESS studies showed a clear curvature improvement in the penile modelling group that is likely greater than placebo alone; this indicates that mechanical/modelling therapies alone may well have some efficacy, as outlined later in this article.

CCH clearly offers a less-invasive approach when compared to surgery; however, it is clear that the degree of curvature improvement is not as significant as is seen with surgery. Nevertheless, it does offer a treatment option for a select group of patients who may not be amenable to surgical intervention and would prefer conservative treatment if possible. The ‘real world’ paper by Anaissie *et al.* succinctly concluded that ultimately CCH needs further study to understand the optimal patient and which treatment regime produces the most efficacious result in terms of curvature improvement without being prohibitive on cost ^42^. We therefore eagerly await the results of larger, multicentre, and randomised studies to further focus on predictive factors for treatment success or failure and provide guidance for implementing CCH treatments in socially funded healthcare systems.

The most recent unexpected update regarding CCH is its untimely removal from the European market by the manufacturers. This is an evolving situation, and the authors hope that an alternative product will be available in the near future ^43^. A summary of the salient studies and most recent clinical evidence in CCH can be found in Table 2.

**Table 2.** Summary of evidence showing the efficacy of CCH.

Author	Year of study	Aim of study	Type of study	No. of patients	Injection protocol	Adjunct	Follow- up (weeks)	Curvature improvement
**Gelbard *et al*. ^36^**	2013	To assess efficacy of CCH alongside penile modelling	RCT	832	Two 0.58 mg injections, 24–72 hours apart Up to eight injections total	Clinician penile modelling	52	17°
**Levine *et al*. ^46^**	2015	To assess efficacy of CCH alongside penile modelling	Phase III open label	347	Two 0.58 mg injections, 24–72 hours apart Up to eight injections total	Clinician penile modelling	36	18.3°
**Ralph *et al*. ^37^**	2017	Assess efficacy of CCH + vacuum therapy +/- penile modelling	Randomised pilot	30	Two 0.58 mg injections, 24–72 hours apart Up to eight injections total	Vacuum therapy +/- penile modelling	36	23.7° in CCH + vacuum therapy + modelling 23.3° in CCH + vacuum therapy
**Abdel Raheem** ***et al*. ^38^**	2017	To assess the safety and efficacy of modified injection protocol	Pilot	53	Three 0.9 mg injections 4 weeks apart	Patient modelling and vacuum therapy	12	17°
**Ziegelmann** ***et al*. ^40^**	2017	To compare efficacy of CCH + traction therapy against CCH alone	Prospective, non- randomised, non-controlled	51	Two 0.58 mg injections, 24–72 hours apart Up to eight injections total	Penile traction 3 hours daily + penile modelling	24	19.6° in CCH and traction 23.6° in CCH alone
**Capece *et al*. ^39^**	2018	To assess efficacy of modified protocol	Multicentre, non- randomised, non-controlled prospective	135	Three 0.9 mg injections 4 weeks apart	Patient modelling and vacuum therapy	12	19°
**Cocci *et al*. ^41^**	2018	To assess the efficacy of CCH + sildenafil	Prospective, non- randomised, non-controlled matched-pair comparison	50	Three 0.9 mg injections 4 weeks apart	Sildenafil 25 mg twice daily prior to patient modelling + vacuum therapy	12	25.6° in CCH + sildenafil 17.4° in CCH alone

CCH, collagenase clostridium histolyticum; RCT, randomised controlled trial

### Verapamil

Intralesional verapamil injections are within the recommendations for treatment in the EUA, AUA, and ICSM guidelines. However, their use is supported by overall poor evidence and hence is given a grade C recommendation by all guidelines ^17– 19^.

Verapamil is a calcium channel blocker that has been shown to interfere with fibroblast proliferation and can decrease collagen deposition by upgrading collagenase activity. There are only two trials comparing verapamil treatment and control subjects.

Rehman *et al.* produced a small randomised control study comparing intralesional verapamil injections against placebo in a group of 14 patients. They used 10–27 mg injections weekly for 6 months. They were able to show a reduction in plaque length, curvature (7.9° in the treatment group compared to 2.2° in the placebo group), and penile pain ^44^. A further RCT by Shirazi *et al.* compared 10 mg verapamil injections to placebo. A total of 80 patients were enrolled and randomised 1:1 to either the treatment or the placebo arm. In the treatment group, patients were given 10 mg injections twice weekly for a total of 12 weeks. In contradiction to Rehman *et al.*, they were unable to find any significant improvement in curvature, plaque size, or penile pain reduction when compared to placebo ^45^.

Favilla *et al.* recently compared intralesional injections of verapamil against hyaluronic acid (HA) in a double blinded randomised study. A total of 132 patients were included and given either weekly 10 mg intralesional verapamil or 16 mg/2 ml injections of HA. Outcomes showed a statistically significant improvement in plaque size (–1.36 mm and –1.8 mm) and IIEF score (1.46 and 1.78) in the verapamil and HA group, respectively. There was no improvement in curvature in the verapamil group in comparison to the HA group, which improved by 4.6° ^47^.

There have been a number of further comparative randomised studies of verapamil over the past 10 years that have significant heterogeneity in their treatment regimens and also their comparative treatments. Greenfield *et al.* compared verapamil with saline in electromotive drug administration (EMDA) for PD. This study failed to show any statistically significant improvement in curvature ^48^. A randomised trial by Mehrsai *et al.* compared treatment in 60 patients treated with verapamil either via injection or EMDA. The authors found no significant difference in reduction of plaque size; however, they did note an improvement in erectile function (albeit not significant). Penile pain assessed by visual analogue scale was significantly reduced in the EMDA group compared with the injection group (–4.1 versus –1.8) at 3 months. Penile curvature was improved in both groups, and increased shift towards a ‘less-than-30°’ group was noted especially in the EMDA category. This improvement, however, was not quantified in more detail or statistically significant ^49^. Abern *et al.* compared verapamil, oral pentoxifylline, and L-arginine (group 1) against the same medical therapy and additional PTT (group 2). Both groups had improved penile curvature, and interestingly the PTT group (group 2) had less curvature improvement (11°) when compared to medical therapy and injections alone (15.1°). The authors did not complete any statistical comparison between the two groups ^50^.

### Interferon α-2B

There have been no recent randomised placebo-controlled studies using interferon (IFN) α-2B. Kendirci *et al.* and Hellstrom *et al.* produced two placebo-controlled studies in 2005 and 2006, respectively. Kendirci *et al.* randomised 5 × 10 ^6^ IU of IFN against placebo with a total of six injections given once weekly. They showed a statistically significant improvement in penile curvature with IFN of 12° compared to 3.6° in the placebo group. Hellstrom *et al.* produced similar results to Kendirci with a similar improvement in curvature ^51, 52^.

More recently, Yafi *et al.* compared IFN with PTT versus IFN alone. This retrospective study had no placebo arm but did show a marginal improvement in curvature of 8.1° in the IFN and traction group compared to 9.9° in the IFN group, respectively. This difference in outcomes between groups was not statistically significant ^53^.

The most recent publication regarding IFN injection therapy was a single-arm prospective non-randomised study performed by Sokhal *et al.* including 86 patients receiving 3 × 10 ^6^ IU of IFN once weekly for a 12-week period. Follow-up was limited to only 3 months; however, there was a statistically significant improvement in plaque size, curvature, and IIEF-5 and pain scores. Plaque length reduced from 12.9 mm to 4.3 mm, and curvature improved by 16.2° (34.8° reduced to 18.6°) over the 3-month treatment period ^54^.

### Hyaluronic acid

Gennaro and colleagues conducted a prospective RCT comparing intralesional HA compared with placebo in 2012. This study comprised 86 patients, with the treatment arm undergoing a total of 30 HA (20 mg) injections over 6 months every 5–7 days. There was a statistically significant improvement in curvature, plaque size, and IIEF when compared to placebo. At 24 months, the curvature was improved by 47% in the treatment arm, which equated to 9°. In the placebo arm, there was a deterioration in curvature of 19° over the same follow-up period ^55^.

A single-arm, multicentre, pilot study was published by Zucchi *et al.* in 2016 and reported on outcomes from a 10-week cycle of HA (16 mg) intralesional injections in a total of 65 patients. They were able to demonstrate a statistically significant improvement in penile plaque length, curvature, IIEF, pain scores, and subjective outcome scores 2 months following the treatment regimen. Curvature was improved by 10° (from 30° to 20°) with no significant adverse events noted throughout the study ^56^.

The comparative study by Favilla *et al.* described previously in this paper also showed advantageous outcomes of HA when compared to verapamil therapy ^47^.

## Mechanical therapy for PD

### Penile traction devices

Mechanical therapy for PD as a treatment modality generally suffers from a lack of robust, randomised controlled studies ^57^. The purported mechanism of action of traction devices is likely to be secondary to cellular mechanotransduction. The mechanical strain on the cell body leads to multiple signal transduction pathways being activated, which is likely to lead to collagen degradation ^58, 59^. They currently have a grade C recommendation by the EAU and ICSM and are not recommended by the AUA owing to a lack of supportive evidence ^17– 19^.

Gontero *et al.* described a regime of increasing mechanical traction (Andropenis® extender device) for a median of 5.5 hours a day over a 6-month period. They were able to produce a marginal non-statistically significant improvement of 4° from 31° to 27° following treatment. There was no improvement in IIEF or penile plaque length; however, there was a statistically significant improvement in stretched penile length of 0.8 cm ^60^.

Using the same device (Andropenis® extender), Martínez-Salamanca and colleagues studied its use in the acute phase of PD over a 6-month period. This prospective non-randomised study compared traction to a non-intervention arm involving patients also considered to be in the acute phase over a 9-month period. In the treatment arm, the prescribed therapy was use of the device for 9 hours per day; however, because of poor adherence, average compliance was 4.6 hours. In the treatment arm, there was a reduction from 33° to 13°, resulting in a 20° curvature improvement. In contrast, the non-intervention arm had worsening curvature over the follow-up period, increasing by 23°. Outcomes were also improved in stretched penile length and IIEF scores in the treatment arm. There was, however, no statistical analysis comparing the two arms of the study, and potentially it is likely that the non-intervention group may have been on several oral therapies for PD ^61^.

The first RCT of mechanical therapy has been published recently by Moncada *et al.* They conducted a study using the PeniMaster® PRO device compared to a control (non-intervention) group with promising results. A total of 93 patients were recruited and assigned to either arm of the study (47 in the treatment group and 43 controls). Follow-up was limited to 3 months, and treatment required use of the device for 3–8 hours daily. There was a clear reduction in penile curvature in the treatment group, which was directly correlated with adherence to device use. Overall reduction was significant at 31.2° (41.1%) compared to baseline, and there was no change in curvature in the control group. This reduction in curvature was enhanced when increasing the usage time. Greater than 6 hours’ use daily resulted in a 36.2° reduction compared to baseline (51.4%), whilst less than 4 hours produced a 19.7° improvement (28.8%). All of these results showed statistical significance. Furthermore, there was also increased stretched penile length and IIEF score. Outcomes overall are promising; however, 43% of patients noted adverse events, which were mainly glans numbness/oedema and local irritation. A total of 6.5% of patients (n = 3) in the treatment arm abandoned the study owing to these adverse events ^62^.

More recently, Ziegelmann *et al.* published a further RCT using a novel mechanical device, RestoreX®. The newer device challenges some of the limitations seen with older, alternative traction devices, namely reduced usage times, increased comfort, and ability to bend the device to exert a focal, non-linear effect. The published trial studied 110 men using the device for only 30–90 minutes a day over a 3-month period. The study group was randomised 3:1 in favour of traction therapy. The results in this trial showed significant promise considering the observed shorter treatment times. A reduction in curvature of 11.7°, improvement in penile length of 1.5 cm, and improvement in IIEF score by 4.3 points were demonstrated ^63^. The control group showed no change in penile length, worsening IIEF, and an increase in curvature by 1.3°. Although this represents a relatively small study with short follow-up time, the results highlight potential positive efficacy and safety outcomes.

Alom *et al.* continued to study the benefits of the RestoreX® device alongside CCH injections ^64^. They performed a comparative cohort study assessing three groups of therapy: CCH alone (group 1, n = 52), CCH plus other mechanical traction devices (group 2, n = 45), and CCH with RestoreX® device (group 3, n = 16). CCH protocol was the same across all patients and used the time schedule described in the IMPRESS trial. However, instead of two injections 24–72 hours per treatment, a single injection of 0.9 mg was used. Various mechanical devices were used in group 2, including the Andropenis® extender as well as the PeniMaster® PRO, which are described earlier in this review. All of these alternative devices required treatment for over 3 hours per day; however, compliance was very poor, with only 16% of this group reaching the required treatment time (median 1.5 hours). In comparison, the RestoreX® group saw a treatment compliance of 97% (median 0.9 hours). The study highlighted an improvement in curvature, which was statistically significant in all groups. Group 1 were able to achieve a 16.5° improvement, group 2 a 20° improvement, and group 3 a 30° improvement. The RestoreX® group also showed a greater improvement in length and various subjective assessments (improved penetration, feeling of meaningful improvement, and estimated percentage improvement) when compared to the other two groups. Overall, the study was able to show the largest degree of curvature change in the literature when compared to any other adjunctive treatment with CCH. It must be noted that these outcomes need to be reproduced in a RCT and in a larger cohort using the RestoreX® treatment group.

The above trials on mechanical therapies have been tabularised in Table 3 with a brief outline of the clinical outcomes.

**Table 3.** Summary of evidence showing the efficacy of mechanical therapies in PD.

Author	Year of study	Aim of study	Type of study	No. of patients	Device	Usage protocol	Follow- up (weeks)	Curvature improvement
Gontero *et al*. ^60^	2009	To assess efficacy of the Andropenis® extender for the treatment of PD	Prospective, non- controlled	15	Andropenis® extender device	5.5 hours daily for 6 months	52	4°
Martínez- Salamanca *et al*. ^61^	2014	To assess efficacy of the Andropenis® extender for the treatment of PD in the acute phase	Prospective, controlled, non- randomised trial	96	Andropenis® extender device	6–9 hours daily for 6 months (4.6 hours/ daily actual compliance)	24	20°
Moncada *et al*. ^62^	2018	To assess the efficacy of the PeniMaster® PRO for the treatment of PD	RCT	93	PeniMaster® PRO	3–8 hours daily for 3 months	12	Mean improvement: 31 ° >6 hours’ use: 36 ° <4 hours’ use: 20°
Ziegelmann *et al*. ^63^	2019	To assess the efficacy of the RestoreX® device for the treatment of PD	RCT	110	RestoreX®	30–90 minutes daily for 3 months	12	11.7°
Alom *et al*. ^64^	2019	To assess the efficacy of CCH +/– traction therapy with either RestoreX® or other devices	Prospective comparative cohort study Group 1 = CCH alone Group 2 = CCH + other devices Group 3 = CCH + RestoreX®	113	RestoreX® or PeniMaster® PRO or Andropenis® extender	30–90 minutes daily for RestoreX® >3 hours daily for other devices (actual use 1.5 hours)	14	Group 1 = 16.5° Group 2 = 20° Group 3 = 30°

CCH, collagenase clostridium histolyticum; PD, Peyronie’s disease; RCT, randomised controlled trial

## External shockwave therapy

There has been limited research on external shockwave therapy (ESWT) for PD in recent times. Its use is not recommended by AUA, EAU, or ICSM guidelines for the treatment of curvature; however, it may have a use for those with penile pain secondary to PD ^17– 19^. A recent meta-analysis by Gao *et al.* reviewed the most recent RCTs using ESWT for PD. They found no statistically significant difference in curvature; however, they did note a statistically significant improvement in pain scores as well as plaque size in the six studies included comprising 443 patients. The authors, however, did note that pain in PD is usually self-limiting, so the role of ESWT in reducing the pain in these patients is arguable ^65^.

## Surgery

### Surgical techniques and outcomes

There have been no significant advances in PD surgical management in terms of randomised controlled data. In keeping with historical trends, there have been numerous retrospective reviews of modifications to traditional plication and grafting techniques. Nevertheless, plication remains the standard of care for patients without erectile dysfunction and a curvature of less than 60° provided that the associated loss of length is not problematic. Incision and grafting are indicated in patients falling outside these criteria, although plaque excision without grafting has been reported previously as a simplified technique ^66^.

Inflatable penile prosthesis (IPP) and manual remodelling have been re-assessed in a retrospective review by Chung *et al.* comparing choice of device manufacturer ^67^. They found high satisfaction (79%) and 5-year mechanical survival (87% or greater) in both AMS CX® and Coloplast Titan® devices as well as comparable revision and complication rates of below 10%. Further results regarding implant insertion and simultaneous plication demonstrated similar levels of satisfaction. Implant surgery remains the mainstay of treatment for patients with significant curvature and erectile dysfunction. Caution remains regarding a distinct lack of evidence to support universal surgical reconstruction in most men in order to provide safe and significant penile lengthening, as advocated by some surgeons globally.

### Graft materials

Graft materials in those undergoing excision of the tunica albuginea in severe PD have been much debated and researched over recent years. The perfect graft material should be traction resistant, be easy to manipulate, and adhere to surrounding tissues with low risk of rejection. It should also be resistant to tension to prevent any aneurysmal dilation during normal erectile function. In addition, it should be economically viable and easily available. There has therefore been a large number of different grafting materials proposed and used in recent research for PD surgery including autografts, allografts, xenografts, and synthetics. Garcia-Gomez *et al.* recently reviewed the current evidence for all graft materials in tunica albuginea excision and grafting procedures. They concluded that the published series has significant heterogeneity in terms of patient selection, outcomes, and follow-up periods, therefore making definitive conclusions difficult. The authors did note that buccal mucosa, pericardium, porcine small intestinal submucosa (SIS), and TachoSil® are being used more extensively than most other materials, but unfortunately there is limited evidence to suggest one material over the other ^68^.

Of note, TachoSil®, which is a fibrin-coated collagen fleece, is becoming an increasingly studied graft material because of its surgical advantages (no requirement to suture graft material). Two comparative studies have reviewed TachoSil® and SIS in penile grafting procedures. Falcone *et al.* compared the graft materials after plaque incision during penile prosthesis implantation in 60 patients. They noted a reduced operative time in the TachoSil® group of 120 minutes compared to 145 minutes in the SIS cohort. There was no difference in functional outcomes or complication rate at 35 months between the two groups ^69^. Rosenhammer *et al.* produced a similar study with a matched-pair analysis to aid in comparative outcome measure. They retrospectively matched 43 patients who underwent penile plaque excision/incision and grafting using SIS with a prospectively collected cohort who had similar demographics and preoperative penile curvature undergoing the same procedure using TachoSil® as the graft material. Once again, operative times were significantly reduced in the TachoSil® group compared to SIS: 80 minutes and 104 minutes, respectively. The TachoSil® group also showed no evidence of recurrence compared to SIS (9%). Shortening was 28% in the SIS group compared to only 5% in the TachoSil® group, which was statistically significant. Complication rates were similar in both groups at under 10% ^70^.

## Future studies and directions

Over recent years, we have observed increasing interest and research into minimally invasive (or non-surgical) treatments for PD. The IMPRESS I and II studies have shaped the clinical landscape promoting the efficacy and safety of intralesional therapy using CCH alongside mechanical therapy devices. Accordingly, we have witnessed a rapid expansion in alternative therapeutic algorithms and improved understanding of the utility of CCH in certain men with PD. Future clinical research should continue to develop uniform research methodology and protocols, including a greater focus on patient-reported outcomes and cost-effectiveness analyses for newer treatments. Whilst multiple researchers have attempted to look into the efficacy of oral therapies for PD, we must concede that there is still no evidence to support their use as first-line treatments in either the acute or the chronic phases. We propose that developing better scientific models may translate into better drug screening and targeting opportunities to realise any meaningful future clinical outcome in RCTs or beyond. To better understand where PD lies among more systemic fibrotic conditions (e.g. normal/aberrant healing, retroperitoneal, liver, or lung fibrosis, etc.) requires careful collaborative efforts to share valuable clinical resources and data using ultimately expensively generated proteomic, genomic, and metabolomic data. This approach should focus on developing new biomarkers, compound screening, and application of improved imaging technology to effectively diagnose, prognose, and treat men with this debilitating heterogeneous and complex problem.

Currently, the future favours an expansion of intralesional CCH usage, becoming the mainstay of treatment for PD if costs and drug availability allow. This depends a lot on engagement from existing pharmaceutical partners, particularly whilst drug patents restrict competition in the open marketplace. There is no doubt that urologists around the world are motivated to help develop and deliver a portfolio of minimally invasive treatments for PD in order to prevent or prolong the time without surgical intervention. Reversing this paradigm will be challenging, but the stepping stones already exist with ‘hotspots’ of high-quality basic scientific and clinical research evidence that will shape future treatments for men with PD. This needs to be matched with an increased general awareness of the problem through public health channels and reflected in a better financial infrastructure to incentivise and reward high-quality research and clinical care.

## Conclusions

Intralesional treatments with modelling are demonstrating notable promise in treating PD non-surgically and at an earlier stage in the disease process. Generally, high treatment costs in non-insurance-based health systems, diverse experience, and the lack of widespread availability impede the current evidence base, particularly in terms of randomised trials.

Whilst CCH outcomes and safety are supported by a large body of published evidence, the ideal treatment regime is still not clear, with a large number of studies being produced without control or comparative arms, thereby making the outcomes difficult to assess. IFN α-2B is the only other injectable therapy worthy of mention at this stage. These studies show a small improvement in curvature with a smaller cohort of patients in comparison to IMPRESS I and II, and the results have not been reproduced for over 10 years. Unfortunately, there has been no further robust research in other injectable intralesional therapies, which will likely limit their translational use.

Penile traction and mechanical devices in PD are very likely to improve outcomes and may well have a further role in the primary treatment of PD or as an adjunct to injectable therapies. Newer penile traction devices show some promise, but further case-control studies will be needed to evaluate their potential as a non-surgical monotherapy ^57^.

Within surgery, there is interest in novel graft materials, and further study into these materials is certainly warranted. In particular, outcomes using TachoSil® when compared to the more commonly used SIS materials have generated interesting data with respect to benefits observed with intraoperative technique and functional outcomes; however, further long-term results and comparative studies are required ^69, 70^.

Currently, we seek to develop improved tools to assess patient depression, systemic health issues, and markers of poor quality of life. PD remains a difficult condition to successfully treat and should remain the remit of dedicated sexual health specialists and surgeons if we are to improve outcomes for men affected by PD and their partners.

## References

[1] Schwarzer U Sommer F Klotz T: The prevalence of Peyronie's disease: results of a large survey. *BJU Int.* 2001;88(7):727–30. 10.1046/j.1464-4096.2001.02436.x 11890244

[2] Mulhall JP Creech SD Boorjian SA: Subjective and objective analysis of the prevalence of Peyronie's disease in a population of men presenting for prostate cancer screening. *J Urol.* 2004;171(6 Pt 1):2350–3. 10.1097/01.ju.0000127744.18878.f1 15126819

[3] Stuntz M Perlaky A des Vignes F: The Prevalence of Peyronie's Disease in the United States: A Population-Based Study. *PLoS One.* 2016;11(2):e0150157. 10.1371/journal.pone.0150157 26907743 PMC4764365

[4] Gelbard MK Dorey F James K: The natural history of Peyronie's disease. *J Urol.* 1990;144(6):1376–9. 10.1016/s0022-5347(17)39746-x 2231932

[5] Mulhall JP Schiff J Guhring P: An analysis of the natural history of Peyronie's disease. *J Urol.* 2006;175(6):2115–8. 10.1016/S0022-5347(06)00270-9 16697815

[6] Baldini A Morel-Journel N Paparel P: Patient-reported long-term sexual outcomes following plication surgery for penile curvature: A retrospective 58-patient study. *Prog Urol.* 2017;27(1):10–6. 10.1016/j.purol.2016.08.018 27867021

[7] Wimpissinger F Parnham A Gutjahr G: 10 Years' Plaque Incision and Vein Grafting for Peyronie's Disease: Does Time Matter? *J Sex Med.* 2016;13(1):120–8. 10.1016/j.jsxm.2015.12.004 26755094

[8] Bilgutay AN Pastuszak AW: Peyronie's Disease: A Review of Etiology, Diagnosis, and Management. *Curr Sex Health Rep.* 2015;7(2):117–31. 10.1007/s11930-015-0045-y 26279643 PMC4535719

[9] Bjekic MD Vlajinac HD Sipetic SB: Risk factors for Peyronie's disease: a case-control study. *BJU Int.* 2006;97(3):570–4. 10.1111/j.1464-410X.2006.05969.x 16469028

[10] El-Sakka AI Selph CA Yen TS: The effect of surgical trauma on rat tunica albuginea. *J Urol.* 1998;159(5):1700–7. 10.1097/00005392-199805000-00097 9554397

[11] De Rose AF Mantica G Bocca B: Supporting the role of penile trauma and micro-trauma in the etiology of Peyronie's disease. Prospective observational study using the electronic microscope to examine two types of plaques. *Aging Male.* 2019;1–6. 10.1080/13685538.2019.1586870 30879382

[12] Milenkovic U Albersen M Castiglione F: The mechanisms and potential of stem cell therapy for penile fibrosis. *Nat Rev Urol.* 2019;16(2):79–97. 10.1038/s41585-018-0109-7 30367131

[13] Gokce A Abd Elmageed ZY Lasker GF: Adipose tissue-derived stem cell therapy for prevention and treatment of erectile dysfunction in a rat model of Peyronie's disease. *Andrology.* 2014;2(2):244–51. 10.1111/j.2047-2927.2013.00181.x 24574095

[14] Gokce A Abd Elmageed ZY Lasker GF: Intratunical Injection of Genetically Modified Adipose Tissue-Derived Stem Cells with Human Interferon α-2b for Treatment of Erectile Dysfunction in a Rat Model of Tunica Albugineal Fibrosis. *J Sex Med.* 2015;12(7):1533–44. 10.1111/jsm.12916 26062100 PMC4914049

[15] Castiglione F Hedlund P van der Aa F: Intratunical injection of human adipose tissue-derived stem cells prevents fibrosis and is associated with improved erectile function in a rat model of Peyronie's disease. *Eur Urol.* 2013;63(3):551–60. 10.1016/j.eururo.2012.09.034 23040209 PMC4029115

[16] Jiang H Gao Q Che X: Inhibition of penile tunica albuginea myofibroblasts activity by adipose-derived stem cells. *Exp Ther Med.* 2017;14(5):5149–5156. 10.3892/etm.2017.5179 29201230 PMC5704342

[17] Hatzimouratidis K Eardley I Giuliano F: EAU guidelines on penile curvature. *Eur Urol.* 2012;62(3):543–52. 10.1016/j.eururo.2012.05.040 22658761

[18] Nehra A Alterowitz R Culkin DJ: Peyronie’s Disease: AUA Guideline. *J Urol.* 2015;194(3):745–53. 10.1016/j.juro.2015.05.098 26066402 PMC5027990

[19] Chung E Ralph D Kagioglu A: Evidence-Based Management Guidelines on Peyronie's Disease. *J Sex Med.* 2016;13(6):905–23. 10.1016/j.jsxm.2016.04.062 27215686

[20] Weidner W Hauck EW Schnitker J: Potassium paraaminobenzoate (POTABA) in the treatment of Peyronie's disease: a prospective, placebo-controlled, randomized study. *Eur Urol.* 2005;47(4):530–6. 10.1016/j.eururo.2004.12.022 15774254

[21] Shah PJR Green NA Adib RS: A multicentre double-blind controlled clinical trial of potassium para-amino-benzoate (POTABA1) in Peyronie’s disease. *Progr Reprod Biol Med J.* 1983;9:61–7.

[22] Park TY Jeong HG Park JJ: The Efficacy of Medical Treatment of Peyronie's Disease: Potassium Para-Aminobenzoate Monotherapy vs. Combination Therapy with Tamoxifen, L-Carnitine, and Phosphodiesterase Type 5 Inhibitor. *World J Mens Health.* 2016;34(1):40–6. 10.5534/wjmh.2016.34.1.40 27169128 PMC4853769

[23] Paulis G Brancato T D'Ascenzo R: Efficacy of vitamin E in the conservative treatment of Peyronie's disease: legend or reality? A controlled study of 70 cases. *Andrology.* 2013;1(1):120–8. 10.1111/j.2047-2927.2012.00007.x 23258640

[24] Paulis G Cavallini G Giorgio GD: Long-term multimodal therapy (verapamil associated with propolis, blueberry, vitamin E and local diclofenac) on patients with Peyronie's disease (chronic inflammation of the tunica albuginea). Results of a controlled study. *Inflamm Allergy Drug Targets.* 2013;12(6):403–9. 10.2174/1871528112666131205112432 24304332

[25] Paulis G Paulis A Romano G: Rationale of combination therapy with antioxidants in medical management of Peyronie's disease: results of clinical application. *Res Rep Urol.* 2017;9:129–39. 10.2147/RRU.S141748 28791261 PMC5530853

[26] Borthwick LA Wynn TA Fisher AJ: Cytokine mediated tissue fibrosis. *Biochim Biophys Acta.* 2013;1832(7):1049–60. 10.1016/j.bbadis.2012.09.014 23046809 PMC3787896

[27] Jalkut M Gonzalez-Cadavid N Rajfer J: New discoveries in the basic science understanding of Peyronie's disease. *Curr Urol Rep.* 2004;5(6):478–84. 10.1007/s11934-004-0074-y 15541219

[28] Gelfand RA Vernet D Kovanecz I: The transcriptional signatures of cells from the human Peyronie's disease plaque and the ability of these cells to generate a plaque in a rat model suggest potential therapeutic targets. *J Sex Med.* 2015;12(2):313–27. 10.1111/jsm.12760 25496134 PMC4339076

[29] Ilg MM Mateus M Stebbeds WJ: Antifibrotic Synergy Between Phosphodiesterase Type 5 Inhibitors and Selective Oestrogen Receptor Modulators in Peyronie's Disease Models. *Eur Urol.* 2019;75(2):329–40. 10.1016/j.eururo.2018.10.014 30344087

[30] Raetsch C Jia JD Boigk G: Pentoxifylline downregulates profibrogenic cytokines and procollagen I expression in rat secondary biliary fibrosis. *Gut.* 2002;50(2):241–7. 10.1136/gut.50.2.241 11788567 PMC1773098

[31] Smith JF Shindel AW Huang YC: Pentoxifylline treatment and penile calcifications in men with Peyronie's disease. *Asian J Androl.* 2010;13(2):322–5. 10.1038/aja.2010.117 21102473 PMC3565600

[32] Alizadeh M Karimi F Fallah MR: Evaluation of verapamil efficacy in Peyronie's disease comparing with pentoxifylline. *Glob J Health Sci.* 2014;6(7 Spec No):23–30. 10.5539/gjhs.v6n7p23 25363175 PMC4796342

[33] Safarinejad MR Asgari MA Hosseini SY: A double-blind placebo-controlled study of the efficacy and safety of pentoxifylline in early chronic Peyronie’s disease. *BJU Int.* 2010;106(2):240–8. 10.1111/j.1464-410X.2009.09041.x 19863517

[34] Ibrahim A Gazzard L Alharbi M: Evaluation of Oral Pentoxifylline, Colchicine, and Penile Traction for the Management of Peyronie's Disease. *Sex Med.* 2019;7(4):459–63. 10.1016/j.esxm.2019.07.003 31445974 PMC6963116

[35] Gelbard MK Walsh R Kaufman JJ: Collagenase for Peyronie's disease experimental studies. *Urol Res.* 1982;10(3):135–40. 10.1007/BF00255956 6291216

[36] Gelbard M Goldstein I Hellstrom WJ: Clinical efficacy, safety and tolerability of collagenase clostridium histolyticum for the treatment of peyronie disease in 2 large double-blind, randomized, placebo controlled phase 3 studies. *J Urol.* 2013;190(1):199–207. 10.1016/j.juro.2013.01.087 23376148

[37] Ralph DJ Abdel Raheem A Liu G: Treatment of Peyronie's Disease With Collagenase *Clostridium histolyticum* and Vacuum Therapy: A Randomized, Open-Label Pilot Study. *J Sex Med.* 2017;14(11):1430–7. 10.1016/j.jsxm.2017.08.015 28974406

[38] Abdel Raheem A Capece M Kalejaiye O: Safety and effectiveness of collagenase clostridium histolyticum in the treatment of Peyronie's disease using a new modified shortened protocol. *BJU Int.* 2017;120(5):717–23. 10.1111/bju.13932 28612401

[39] Capece M Cocci A Russo G: Collagenase clostridium histolyticum for the treatment of Peyronie's disease: A prospective Italian multicentric study. *Andrology.* 2018;6(4):564–7. 10.1111/andr.12497 29733116

[40] Ziegelmann MJ Viers BR Montgomery BD: Clinical Experience With Penile Traction Therapy Among Men Undergoing Collagenase *Clostridium histolyticum* for Peyronie Disease. *Urology.* 2017;104:102–9. 10.1016/j.urology.2017.01.054 28347795

[41] Cocci A Cito G Urzì D: Sildenafil 25 mg ODT + Collagenase *Clostridium hystoliticum* vs Collagenase *Clostridium hystoliticum* Alone for the Management of Peyronie’s Disease: A Matched-Pair Comparison Analysis. *J Sex Med.* 2018;15(10):1472–7. 10.1016/j.jsxm.2018.08.012 30245025

[42] Anaissie J Hellstrom WJG Yafi FA: Collagenase *Clostridium Histolyticum* for the Treatment of Peyronie’s Disease: A ‘Real World’ Clinical Perspective. *Drugs.* 2016;76(16):1523–8. 10.1007/s40265-016-0649-1 27770352

[43] Cocci A Russo GI Salamanca JIM: The End of an Era: Withdrawal of Xiapex ( *Clostridium histolyticum Collagenase*) from the European Market. *Eur Urol.* 2020;77(5):660–1. 10.1016/j.eururo.2019.11.019 31810821

[44] Rehman J Benet A Melman A: Use of Intralesional Verapamil to Dissolve Peyronie’s Disease Plaque: A Long-Term Single-Blind Study. *Urology.* 1998;51(4):620–6. 10.1016/s0090-4295(97)00700-0 9586617

[45] Shirazi M Haghpanah AR Badiee M: Effect of intralesional verapamil for treatment of Peyronie's disease: A randomized single-blind, placebo-controlled study. *Int Urol Nephrol.* 2009;41(3):467–71. 10.1007/s11255-009-9522-4 19199072

[46] Levine LA Cuzin B Mark S: Clinical safety and effectiveness of collagenase clostridium histolyticum injection in patients with Peyronie's disease: A phase 3 open-label study. *J Sex Med.* 2015;12(1):248–58. 10.1111/jsm.12731 25388099

[47] Favilla V Russo GI Zucchi A: Evaluation of intralesional injection of hyaluronic acid compared with verapamil in Peyronie's disease: Preliminary results from a prospective, double-blinded, randomized study. *Andrology.* 2017;5(4):771–5. 10.1111/andr.12368 28718527

[48] Greenfield JM Shah SJ Levine LA: Verapamil versus saline in electromotive drug administration for Peyronie's disease: A double-blind, placebo controlled trial. *J Urol.* 2007;177(3):972–5. 10.1016/j.juro.2006.10.065 17296390

[49] Mehrsai AR Namdari F Salavati A: Comparison of transdermal electromotive administration of verapamil and dexamethasone versus intra-lesional injection for Peyronie's disease. *Andrology.* 2013;1(1):129–32. 10.1111/j.2047-2927.2012.00018.x 23258641

[50] Abern MR Larsen S Levine LA: Combination of penile traction, intralesional verapamil, and oral therapies for Peyronie's disease. *J Sex Med.* 2012;9(1):288–95. 10.1111/j.1743-6109.2011.02519.x 22024053

[51] Kendirci M Usta MF Matern RV: The impact of intralesional interferon alpha-2b injection therapy on penile hemodynamics in men with Peyronie's disease. *J Sex Med.* 2005;2(5):709–15. 10.1111/j.1743-6109.2005.00110.x 16422829

[52] Hellstrom WJG Kendirci M Matern R: Single-blind, multicenter, placebo controlled, parallel study to assess the safety and efficacy of intralesional interferon alpha-2B for minimally invasive treatment for Peyronie's disease. *J Urol.* 2006;176(1):394–8. 10.1016/S0022-5347(06)00517-9 16753449

[53] Yafi FA Pinsky MR Stewart C: The Effect of Duration of Penile Traction Therapy in Patients Undergoing Intralesional Injection Therapy for Peyronie's Disease. *J Urol.* 2015;194(3):754–8. 10.1016/j.juro.2015.03.092 25804087

[54] Sokhal A Jain N Jhanwar A: Prospective study to evaluate the clinical outcome of intralesional interferon-α 2b in the management of Peyronie's disease. *Urol Ann.* 2018;10(2):154–158. 10.4103/UA.UA_65_17 29719326 PMC5907323

[55] Gennaro R Barletta D Paulis G: Intralesional hyaluronic acid: An innovative treatment for Peyronie's disease. *Int Urol Nephrol.* 2015;47(10):1595–602. 10.1007/s11255-015-1074-1 26257044

[56] Zucchi A Costantini E Cai T: Intralesional Injection of Hyaluronic Acid in Patients Affected With Peyronie's Disease: Preliminary Results From a Prospective, Multicenter, Pilot Study. *Sex Med.* 2016;4(2):e83–8. 10.1016/j.esxm.2016.01.002 26984291 PMC5005307

[57] Russo GI Milenkovic U Hellstrom W: Clinical Efficacy of Injection and Mechanical Therapy for Peyronie's Disease: A Systematic Review of the Literature. *Eur Urol.* 2018;74(6):767–81. 10.1016/j.eururo.2018.07.005 30237020

[58] Chung E de Young L Solomon M: Peyronie's Disease and Mechanotransduction: An In Vitro Analysis of the Cellular Changes to Peyronie's Disease in a Cell-Culture Strain System. *J Sex Med.* 2013;10(5):1259–67. 10.1111/jsm.12082 23421851

[59] Setia SA Levine LA: Devices for penile traction: The long and winding road to treating Peyronie's disease. *Expert Rev Med Devices.* 2018;15(8):517–26. 10.1080/17434440.2018.1502083 30016597

[60] Gontero P Di Marco M Giubilei G: Use of Penile Extender Device in the Treatment of Penile Curvature as a Result of Peyronie's Disease. Results of a Phase II Prospective Study. *J Sex Med.* 2009;6(2):558–66. 10.1111/j.1743-6109.2008.01108.x 19138361

[61] Martínez-Salamanca JI Egui A Moncada I: Acute phase Peyronie's disease management with traction device: A nonrandomized prospective controlled trial with ultrasound correlation. *J Sex Med.* 2014;11(2):506–15. 10.1111/jsm.12400 24261900

[62] Moncada I Krishnappa P Romero J: Penile traction therapy with the new device ‘Penimaster PRO’ is effective and safe in the stable phase of Peyronie's disease: A controlled multicentre study. *BJU Int.* 2019;123(4):694–702. 10.1111/bju.14602 30365247

[63] Ziegelmann M Savage J Toussi A: Outcomes of a Novel Penile Traction Device in Men with Peyronie's Disease: A Randomized, Single-Blind, Controlled Trial. *J Urol.* 2019;202(3):599–610. 10.1097/JU.0000000000000245 30916626

[64] Alom M Sharma KL Toussi A: Efficacy of Combined Collagenase Clostridium histolyticum and RestoreX Penile Traction Therapy in Men with Peyronie’s Disease. *J Sex Med.* 2019;16(6):891–900. 10.1016/j.jsxm.2019.03.007 30956106

[65] Gao L Qian S Tang Z: A meta-analysis of extracorporeal shock wave therapy for Peyronie’s disease. *Int J Impot Res.* 2016;28(5):161–6. 10.1038/ijir.2016.24 27250868

[66] Mantovani F Patelli E Antolini C: Peyronie's disease: Endocavernous plaque excision without substitutive graft: critical 5-year experience. *Urologia.* 2013;80(Suppl 22):28–30. 10.5301/RU.2013.10599 23334885

[67] Chung PH Francis Scott J Morey AF: High Patient Satisfaction of Inflatable Penile Prosthesis Insertion with Synchronous Penile Plication for Erectile Dysfunction and Peyronie's Disease. *J Sex Med.* 2014;11(6):1593–8. 10.1111/jsm.12530 24708140

[68] Garcia-Gomez B Ralph D Levine L: Grafts for Peyronie's disease: A comprehensive review. *Andrology.* 2018;6(1):117–26. 10.1111/andr.12421 29266877

[69] Falcone M Preto M Ceruti C: A Comparative Study Between 2 Different Grafts Used as Patches After Plaque Incision and Inflatable Penile Prosthesis Implantation for End-Stage Peyronie's Disease. *J Sex Med.* 2018;15(7):1061–1062. 10.1016/j.jsxm.2018.04.646 29861353

[70] Rosenhammer B Sayedahmed K Fritsche HM: Long-term outcome after grafting with small intestinal submucosa and collagen fleece in patients with Peyronie's disease: A matched pair analysis. *Int J Impot Res.* 2019;31(4):256–62. 10.1038/s41443-018-0071-1 30194372

